# Hierarchical Multi-Scale Coupled Periodical Photonic and Plasmonic Nanopatterns Inscribed by Femtosecond Laser Pulses in Lithium Niobate

**DOI:** 10.3390/nano12234303

**Published:** 2022-12-04

**Authors:** Sergey Kudryashov, Alexey Rupasov, Mikhail Kosobokov, Andrey Akhmatkhanov, George Krasin, Pavel Danilov, Boris Lisjikh, Alexander Abramov, Evgeny Greshnyakov, Evgeny Kuzmin, Michael Kovalev, Vladimir Shur

**Affiliations:** 1 Lebedev Physical Institute, 119991 Moscow, Russia; 2 School of Natural Sciences and Mathematics, Ural Federal University, 620000 Ekaterinburg, Russia

**Keywords:** lithium niobate, femtosecond laser, bulk inscription, hierarchical longitudinal and transverse nanogratings, standing electromagnetic and ionization waves, interference of interfacial plasmons

## Abstract

The ultrafast interaction of tightly focused femtosecond laser pulses with bulk dielectric media in direct laser writing (inscription) regimes is known to proceed via complex multi-scale light, plasma and material modification nanopatterns, which are challenging for exploration owing to their mesoscopic, transient and buried character. In this study, we report on the first experimental demonstration, analysis and modeling of hierarchical multi-period coupled longitudinal and transverse nanogratings in bulk lithium niobate inscribed in the focal region by 1030 nm, 300 fs laser pulses in the recently proposed sub-filamentary laser inscription regime. The longitudinal Bragg-like topography nanogratings, possessing the laser-intensity-dependent periods ≈ 400 nm, consist of transverse birefringent nanogratings, which are perpendicular to the laser polarization and exhibit much smaller periods ≈ 160 nm. Our analysis and modeling support the photonic origin of the longitudinal nanogratings, appearing as prompt electromagnetic and corresponding ionization standing waves in the pre-focal region due to interference of the incident and plasma-reflected laser pulse parts. The transverse nanogratings could be assigned to the nanoscale material modification by interfacial plasmons, excited and interfered in the resulting longitudinal array of the plasma sheets in the bulk dielectric material. Our experimental findings provide strong support for our previously proposed mechanism of such hierarchical laser nanopatterning in bulk dielectrics, giving important insights into its crucial parameters and opening the way for directional harnessing of this technology.

## 1. Introduction

Flexible ultrashort-pulse laser nanopatterning of bulk dielectrics appears as a key enabling technology for next generations of all-dielectric metamaterial platforms made of multiple stacks of different functional metasurfaces. Though these opportunities for versatile nanoscale light control and manipulation are still emerging, various laser nanopatterning modalities have already been developed to produce in dielectric media high-contrast refractive index structures based on atomistic densification [1,2], two-photon polymerization [3], nano-ablation [4,5], periodic nanoscale material self-organization and form-birefringence [6,7,8] for well-established applications in direct laser writing (inscription) of light waveguides [9] and more complex functional morphologies [2], hollow optical memory bits [4], microfluidic channels [10] and polarizing optical elements and devices [11,12,13]. Meanwhile, novel promising nanopatterning modalities based on delicate and precise ultrashort-pulse (femtosecond or picosecond, fs/ps) laser inscription in bulk dielectrics are still under intense scientific studies, being highly challenging for accurate fabrication and informative characterization on this dielectric platform because of their mesoscopic, transient and buried character.

**Figure 1:**
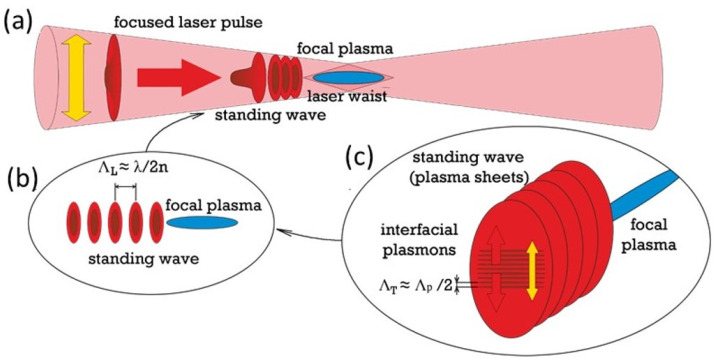
(**a**) Schematic of fs-laser-induced focal plasma and pre-focal electromagnetic/ionization standing waves (yellow arrow—laser polarization direction). (**b**) Structure of the standing wave of electromagnetic field and plasma sheets. (**c**) Transverse plasmonic sub-structure of the plasma sheet array.

**Figure 2:**
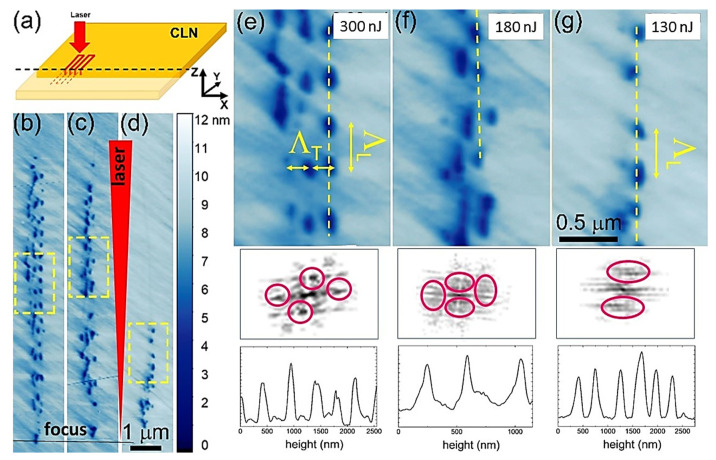
(**a**) Optical layout of fs-laser inscription, CLN sample arrangement and its cross cut for AFM characterization. (**b**–**d**) Panoramic AFM images of the uncovered longitudinal (vertical) and transverse (horizontal) nanopatterned relief topographies, inscribed at different fs-laser pulse energies of 130, 180 and 300 nJ, respectively. (**e**–**g**) (top) Magnified views of the characteristic transverse nanopattern topographies highlighted in (**b**–**d**) by yellow dashed frames; (bottom) their 2D FFT spectra and longitudinal relief profiles along the yellow dashed lines in (**e**–**g**).

**Figure 3:**
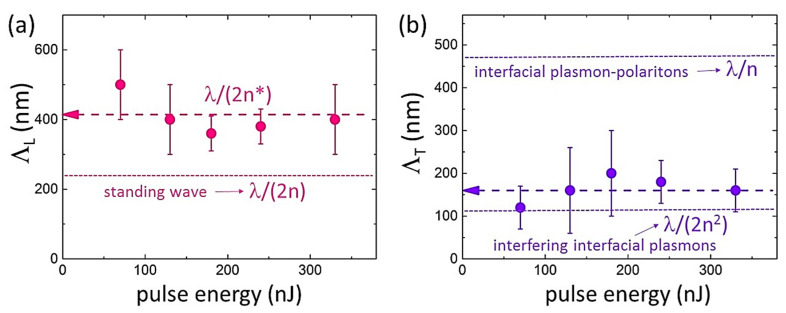
(**a**) Periods of longitudinal (Λ_L_, violet symbols) and (**b**) transverse (Λ_T_, pink symbols) nanopatterns versus fs-laser pulse energy. The corresponding colored dashed lines indicate the average Λ_L_ and Λ_T_ magnitudes, while dotted lines indicate the expected longitudinal nanopattern period λ/(2n) ≈ 235 nm in (**a**), and the expected transverse nanopattern periods for interfacial plasmon-polaritons λ/n ≈ 470 nm and for interfering interfacial plasmons λ/(2n^2^) ≈ 110 nm in (**b**).

**Figure 4:**
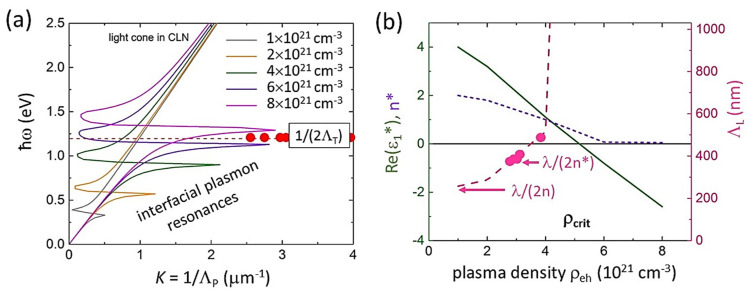
(**a**) Color dispersion curves ħω-K of interfacial plasmons in CLN at different e-h plasma densities in the range of (1–8) × 10^21^ cm^−3^ and mapping of the plasmon resonance at 1030 nm (1.2 eV), using the experimental 1/(2Λ_T_) values (red circles). (**b**) Left axis: dependences of Re(ε*) (green curve) and n* (violet dot curve) at 1030 nm on ρ_eh_; right axis: calculated periods λ/(2n*) (pink dashed curve) in comparison to experimental values Λ_L_ (pink circles) versus ρ_eh_.

Recently, a new ultrashort-pulse laser inscription modality was proposed for hierarchical nanopatterning of bulk dielectrics [14,15], via self-organization of birefringent nanograting arrays (Figure 1), utilizing the flexible combinations of laser wavelengths λ, pulsewidths τ, pulse energies E, focusing conditions and diverse dielectric materials—fluorite and fused silica [15,16]. Very surprisingly, such birefringent nanopatterns highly extended along the laser beam waist were observed in a linear (sub-filamentary) focusing regime [14,15,16], rather than along the extended non-linear (filamentary) focus of ultrashort laser pulses, possessing peak powers well above the critical one for Kerr self-focusing [7,17,18]. The self-organized hierarchical nanopatterns—periodical sub-wavelength longitudinal stacks of transverse nanogratings—were assumed to proceed via four schematic main steps: (1) formation of reflective electron-hole plasma of near-critical density in the linear focus (Figure 1a); (2) longitudinal interference of the reflected and incident linearly polarized pulse parts in the pre-focal region, formation of the near-plane standing electromagnetic wave and the corresponding ionization wave (plasma sheets) with the period Λ_L_ ≈ λ/(2n) (*photonic nanostructure*) [14,15] (Figure 1a,b); (3) excitation and interference of interfacial (boundary between weakly/strongly photoexcited dielectric layers) sub-wavelength plasmons (wavelength Λ_P_ ~ λ/n^2^ ≪ λ [19]), counter-propagating along or normal to the laser polarization [20] in the pre-focal stack of the near-plane plasma sheets separated by the distance Λ_L_ (Figure 1c); (4) periodical structural modification of the dielectric material and the corresponding modulation of the refractive index in the standing electromagnetic/ionization wave of the interfering plasmons (period Λ_T_ ≈ Λ_P_/2, *ultrafine plasmonic sub-structure of the photonic one*) [19,21]. Although elaborate and comprehensive visualization of bulk nano- and microscale patterns produced by ultrashort laser pulses in dielectric media has been performed to the date in a limited number of studies [6,7,8,12,17,18,22], the assignment of patterns to sub-filamentary or filamentary laser focusing regimes, as well as the detailed topographic analysis of the internal structure as a function of laser wavelength, pulse width and energy/intensity/peak power, is still missing, while the visualization results in different studies are quite contradictory in their pulse energy/power trends. Specifically, comprehensive ultrafine visualization was not performed yet for the abovementioned sub-filamentary ultrashort-pulse laser nanopatterning regime to envision the predicted well-ordered hierarchical nanopatterns [14,15]. Moreover, this new ultrashort-pulse laser hierarchical nanopatterning modality is of interest for inscription of functional nanostructures in other, still unexplored dielectric materials (e.g., polymers, ferroelectrics, etc. [23,24,25,26,27]) regarding its relevance and universal performance. Finally, ultrafine visualization analysis could facilitate new emerging functional applications of the predicted hierarchical nanopatterns in nano-optics, quantum and non-linear optics and material science.

In this study, we present for the first time ultrafine comparative experimental visualization, analysis and modeling of multi-scale internal nano-topographies of hierarchical coupled longitudinal photonic and transverse plasmonic nanopatterns in bulk lithium niobate inscribed in the focal region by tightly focused 1030 nm, 300 fs laser pulses in the sub-filamentary laser inscription regime. Our analysis reveals the slight diminishing laser intensity/plasma density effect on the spatial periods of the *photonic* longitudinal nanopatterns accompanying the standing electromagnetic/ionization waves. Likewise, the total height and stripe number of *plasmonic* transverse birefringent nanogratings increase versus laser intensity, ionizing broader beam aperture across and along the optical path in the waist. These findings enable flexible managing of the internal hierarchical nano-topographies by broad tuning of ultrashort-pulse laser parameters for emerging functional applications.

## 2. Materials and Methods

In these studies, we used a congruent lithium niobate (CLN) crystalline z-cut plate with its spontaneous polarization parallel to the z-axis (Figure 2a). For laser-induced bulk nanopatterning of the CLN crystal, a 3D-micro/nanostructuring laser workstation based on the femtosecond Yb-doped fiber laser system Satsuma (Amplitude Systemes, France) with the fundamental wavelength λ = 1030 nm (TEM_00_), full-width at half-maximum pulse duration τ ≈ 300 fs, variable pulse energy *E* ≤ 10 μJ and repetition rate *f* = 0–500 kHz was employed. The laser pulses were focused by a 0.65 NA micro-objective lens into the 1/e-intensity radius w_0_ = 1.2 ± 0.1 μm at the depth ~60 µm inside the lithium niobate crystal. The sample was mounted on a PC-driven high-precision three-dimensional (XYZ) motorized micro-positioning translation stage (Prior Scientific, UK) and scanned at the translation speed of 400 μm/s, enabling inscription at different delivered energies *E* = 50–300 nJ (peak power *P* ≈ 0.17–1.0 MW and peak fluence *F* ≈ 1–8 J/cm^2^) in the sub-filamentary (linear focusing) regime (*P* ≤ *P*_crit_ = 0.9 ± 0.1 MW for the critical Kerr self-focusing power for z-cut CLN at 1030 nm [23]) 3 mm wide line arrays (series of 3 lines each) with the 3 µm inter-line spacing and the scan direction along the laser polarization.

In order to reveal the ultrafine nano-topography of the buried fs-laser nanopatterned CLN regions, the inscribed linear horizontal arrays of vertical nanopatterns in the bulk CLN were saw-cut across the scan lines by an automated precision dicing saw DAD 3220 (DISCO, Japan), using a Disco diamond blade disk Z09-SD3000-Y1-90 55x0.1 A2X40-L (DISCO). The cuts were consequently grinded by Al_2_O_3_ powders (grain sizes: 30, 9 and 3 μm) and polished by ≈25 nm colloidal SiO_2_ nanoparticles on the polishing machine PM5 (Logitech, UK) until optical surface quality. Then, the uncovered topography was characterized by an atomic force microscope NTEGRA Aura (NT-MDT, Russia) in the piezoelectric response mode, using Pt-coated NSC 18 probes (MikroMash, Russia, tip size—30 nm, first resonance frequency—400–500 kHz and stiffness coefficient—2.8 N/m) at the 10-V, 20-kHz probing ac voltage. The acquired surface topographies inscribed at the different fs-laser pulse energies (fluences) are presented in Figure 2b–d along with their two-dimensional (2D) fast Fourier transform (FFT) spectra and longitudinal relief profiles (Figure 2e–g).

## 3. Experimental Results and Discussion

These hierarchical well-organized buried nanopattern topographies, observed for the first time in comparison to laser-inscribed single periodical nano- or micropatterns [6,7,8,12,17,18,22], exhibit the ultrafine periodical longitudinal pre-focal structure, composed by the periodical transverse one, with the typical nanorelief height ~10 nm (Figure 2b–d). In the linear focusing regime, at the higher fs-laser pulse energies the length, *L*, of the ablatively nanopatterned regions appears larger (the larger pre-focal high-intensity/fluence region above the local ablation threshold) with the larger number of longitudinal nanopatterns *N*, but with the almost constant sub-wavelength period Λ_L_ = *L*/*N* ≈ 400 nm (Figure 2a and Figure 3a). Likewise, the number of the transverse nanopatterns increases versus increasing *E* (Figure 2b–f), exhibiting also the almost constant but even smaller sub-wavelength period Λ_T_ ≈ 160 nm (Figure 2b and Figure 3b). For the same reason, the longitudinal nanopatterns become sharper (less transverse nanopatterns) when farther from the laser focus, with some nano-features less pronounced or stacking faults in the transverse nanopatterns between the neighboring longitudinal stripes. These trends are perfectly consistent with the threshold appearance of the ablative nanopatterns at the fs-laser pulse energies, exceeding the overall ablation threshold pulse energy value ≈50 nJ (fluence—1.2 J/cm^2^). Furthermore, similar to other sub-filamentary fs-laser-inscribed birefringent nanopatterns in dielectrics [14,15,16], the nanopatterned regions in CLN exhibit high pulse-energy tunable retardance magnitudes up to λ/5, measured by a birefringence imaging system Thorlabs LCC7201B (not shown).

Surprisingly, both the measured longitudinal photonic and transverse plasmonic nanopattern periods Λ_L_, Λ_T_ exhibit deeply sub-wavelength scales, while the former quantity (≈400 nm) considerably differs from the expected magnitude λ/(2n) ≈ 235 nm (where n ≈ 2.15 is the ordinary wave refractive index value at the 1030 nm wavelength in CLN [28]) for the standing electromagnetic wave in the bulk dielectric. This effect could be associated with the spatially continuous prompt electron-hole plasma (EHP) diminishing of the real part of the dielectric function in the photoexcited CLN, Re[ε*] (and the corresponding refractive index value n*), along the pre-focal laser interference region. To obtain insight into the intriguing sub-wavelength scales of the longitudinal and transverse nanopattern periods, Λ_L_ and Λ_T_, respectively, the prompt dielectric function of the photoexcited CLN was modeled as a function of EHP density ρ_eh_ and optical frequency Ω in the following form [29]:

(1) $$\varepsilon^{*}\left(\Omega ,\rho_{eh}\right)=\varepsilon \left(\Omega \right)\left(1-\frac{\rho_{eh}}{\rho_{sat}}\right)-\frac{\Omega_{PL}^{2}(\rho_{eh})}{\Omega_{}^{2}+\nu {\left(\rho_{eh}\right)}^{2}}\left(1-\frac{i\nu (\rho_{eh})}{\Omega }\right)$$

where the EHP frequency Ω_PL_(ρ_eh_) and scattering rate ν(ρ_eh_) were evaluated as follows [21]:

(2) $$\Omega_{PL}^{2}(\rho_{eh})=\frac{\rho_{eh}e^{2}}{\varepsilon_{0}\varepsilon_{\mathrm{hf}}(\rho_{eh})m_{opt}^{*}},\;\nu (\Omega ,\rho_{eh})=\left(\frac{\pi^{2}\sqrt{3}}{128E_{F}{}^{2}}\right)\frac{{\left(\pi k_{B}T_{e}\right)}^{2}+{\left(\hbar \Omega \right)}^{2}}{1+\exp\left(\frac{-\hbar \Omega }{k_{B}T_{e}}\right)}\Omega_{PL}(\rho_{eh})\propto C\Omega_{PL}(\rho_{eh}{)}^{,}$$

accounting for the effective optical EHP mass *m*_opt_*, the high-frequency dielectric constant ε_hf_(ρ_eh_) due to EHP screening tending to 1 at near-critical EHP densities ρ_eh_ ~ρ_crit_ (ρ_crit_ ≈ 5 × 10^21^ cm^−3^ in CLN at 1030-nm wavelength, Figure 4b), defined from Equation (1) as Ω_PL_(ρ_crit_) = $\sqrt{\varepsilon (\Omega )}$ Ω, EHP saturation density for interband transitions ρ_sat_, temperature *T*_e_ and Fermi level *E*_F_ and the numerical factor *C* ~10 in different dielectrics [15,19,21,30]. See the other calculation details in Supplementary Materials.

Then, the dielectric function ε*(Ω, ρ_eh_) was used to evaluate the wavenumber *K* = 1/Λ_P_ of plasmons, propagating at the “weakly/strongly photoexcited CLN” interface of the plasma sheets in the pre-focal region, through the common dispersion relationship for surface (here—interfacial, IPP) plasmon-polaritons [31]:

(3) $$K=ℜe\left(\frac{1}{\lambda }\sqrt{\frac{\varepsilon *\varepsilon }{\varepsilon *+\varepsilon }}\right)$$

where the complex dielectric functions of the photoexcited and unexcited CLN are ε*(Ω, ρ_eh_) and ε(Ω), respectively. The results of our calculations of IPP dispersion curves, utilizing the complete form of Equation (3) [32], which was previously successfully applied in SPP simulations for different metals, semiconductors and dielectrics immersed in various dielectric media [15,19,33], are given in Figure 4a.

The calculated dispersion curves demonstrate a series of the interfacial plasmon resonances, raising in their energy versus the increasing EHP density ρ_eh_ = (1–8) × 10^21^ cm^−3^ (Figure 4a). Particularly, the plasmon resonance approaches 1.2-eV energy (laser wavelength—1030 nm) at the densities (6–8) × 10^21^ cm^−3^, where ε*(1.2 eV, ρ_eh_ > ρ_crit_) ≈ −ε(1.2 eV) as the basic requirement for the interfacial plasmonic resonance [31]. The experimental data values 1/(2Λ_T_) are reasonably mapping the IPP resonances in Figure 4a; moreover, these experimental values Λ_T_ are also reasonably consistent with the transverse nanopattern periods, the expected periods for interfering undamped interfacial plasmons λ/(2n^2^) ≈ 110 nm [19] (Figure 3b), rather than for interfacial plasmon-polaritons λ/n ≈ 470 nm.

Finally, these simulations enable to evaluate the quasi-continuous component of the EHP density in the pre-focal laser interference region, which decreases the local Re(ε*) and n* magnitudes to result in the longitudinal nanopattern periods λ/(2n*) ≈ 400 nm, rather than λ/(2n) ≈ 235 nm (Figure 3a and Figure 4b). The evaluated EHP density (Figure 4b) is reasonably subcritical, ρ_eh_ ≈ 3 × 10^21^ cm^−3^ < ρ_crit_, to enable microscale laser penetration and interference in the plasma.

## 4. Conclusions

In this study, hierarchical nanopatterning by ultrashort laser pulses was for the first time observed in the bulk dielectric material (crystalline lithium niobate) in the linear focusing (sub-filamentary) regime, exhibiting the longitudinal *photonic* nanopatterns with the period of ≈ 400 nm, consisting of transverse *plasmonic* nanopatterns with the period of ≈ 160 nm. The related analysis and modeling indicate the interference (standing wave) photonic origin of the longitudinal nanopatterns in the pre-focal sub-critical electron-hole plasma, while in the interference maxima plasma density approaches near-critical magnitudes, supporting excitation of deeply sub-wavelength interfacial plasmons at the dielectric/plasma interfaces. These results provide important insights into our previously proposed mechanism of such hierarchical sub-filamentary laser nanopatterning in bulk dielectrics and uncover novel opportunities of its advanced applications in nano-optics, quantum and non-linear optics and material science.

## Data Availability

The data supporting the reported results are accompanying this submission.

## Supplementary Materials

The following supporting information can be downloaded at: https://www.mdpi.com/article/10.3390/nano12234303/s1, References [34,35,36,37,38,39] are cited in the Supplementary Materials.
