# Supplementary material for: Hierarchical Multi-Scale Coupled Periodical Photonic and Plasmonic Nanopatterns Inscribed by Femtosecond Laser Pulses in Lithium Niobate
Source: Nanomaterials (Basel). 2022 Dec 4;12(23):4303. doi: 10.3390/nano12234303 (PMC9739945; doi:10.3390/nano12234303)
Supplement: Supplementary file 1 [file nanomaterials-12-04303-s001.zip › nanomaterials-2053317-supplementary.pdf]

SUPPORTING INFORMATION

Article

Hierarchical Multi-Scale Coupled Periodical Photonic and Plasmonic Nanopatterns Inscribed by Femtosecond Laser Pulses in Lithium Niobate

Sergey Kudryashov, [1,2]* Alexey Rupasov, [1] Mikhail Kosobokov, [2] Andrey Akhmatkhanov, [2] George Krasin, [1] Pavel Danilov, [1,2] Boris Lisjikh, [2] Alexander Abramov, [2] Evgeny Greshnyakov, [2] Evgeny Kuzmin, [1] Michael Kovalev and [1,2] Vladimir Shur [2]

[1] Lebedev Physical Institute, 119991 Moscow, Russia
[2] School of Natural Sciences and Mathematics, Ural Federal University, Ekaterinburg 620000, Russia
* Correspondence: kudryashovsi@lebedev.ru

In general case, wavenumbers of surface (interfacial) plasmon-polaritons (SPP,I PP) on the metallic (photo-excited) surface, neighboring with a dielectric medium, could be calculated according to the common SPP dispersion relationship [1]

$$K_{SPP} = \Re e\left(\frac{1}{\lambda}\sqrt{\frac{\varepsilon_M \varepsilon_D}{\varepsilon_M + \varepsilon_D}}\right), \qquad \text{(S1)}$$

for the dielectric permittivities $\varepsilon_M = \Re e(\varepsilon_M) + i\Im m(\varepsilon_M) = \varepsilon_1 + i\varepsilon_2$ of the photoexcited (metallized) material and $\varepsilon_D = \Re e(\varepsilon_D) + i\Im m(\varepsilon_D) = \eta_1 + i\eta_2$ of the contact solid, liquid, or gaseous dielectric. Extremely short nanoripple periods can be achieved under surface/interface plasmon resonance (SPR) conditions via normal-incidence excitation of counterpropagating ultimately short-wavelength surface plasmons at $|\Re e(\varepsilon_M)|$ tending to $\Re e(\varepsilon_D)$, where in the SPP excitation regime $\Re e(\varepsilon_M) < 0$ and $\Re e(\varepsilon_D) > 0$. The corresponding plasmonic interference results in a standing surface EM pattern with its periodicity of $1/(2K_\infty)$ [2-9]. In SPR one has $\Re e(\varepsilon_M) = -\Re e(\varepsilon_d)$, making its peak wavenumber K_∞ and width dependent only on $\Im m(\varepsilon_M)$, while the exact calculations of K_∞ are going beyond the common approximation $\Re e(\varepsilon_M) \ll -\Re e(\varepsilon_D)$, $|\Re e(\varepsilon_M)| \gg \Im m(\varepsilon_M)$ with its simplified resulting expression for $K_{SPP} = \Re e\left(\frac{1}{\lambda}\sqrt{\varepsilon_D}\right) = \frac{n_D}{\lambda}$ [10].

More accurately, K_∞ magnitudes were calculated, using in the SPR description the exact, complete SPP dispersion equations as follows [11]

$$K_{SPP} = \frac{1}{\lambda}\sqrt{\frac{b+\sqrt{b^2+c^2}}{2a}}, \qquad \text{(S2)}$$

where the factors a, b and c are defined in the form

$$a = (\eta_1+\varepsilon_1)^2 + (\eta_2+\varepsilon_2)^2.$$
$$b = \eta_1(\varepsilon_1^2+\varepsilon_2^2) + \varepsilon_1(\eta_1^2+\eta_2^2), \qquad \text{(S3)}$$
$$c = \eta_2(\varepsilon_1^2+\varepsilon_2^2) + \varepsilon_2(\eta_1^2+\eta_2^2).$$

In the SPR, where $\varepsilon_1 = -\eta_1$ for the resonance condition $\Re e(\varepsilon_m) = -\Re e(\varepsilon_d)$, for $\eta_2 \approx 0$ [12]

$$a = \varepsilon_2{}^2, \quad b = \eta_1\varepsilon_2^2, \quad c = \varepsilon_2\eta_1^2, \qquad \text{(S4)}$$

and for $b \ll c$ at $\varepsilon_2 \ll 1 < \{|\varepsilon_1|, \eta_1\}$

$$K_\infty = \frac{1}{\lambda}\sqrt{\frac{b+\sqrt{b^2+c^2}}{2a}} \approx \frac{1}{\lambda}\sqrt{\frac{c}{2a}} \approx \frac{\sqrt{\eta_1^2\varepsilon_2}}{\lambda\sqrt{2\varepsilon_2^2}} \propto \frac{n_D^2}{\lambda}\frac{1}{\sqrt{2\varepsilon_2}}, \qquad \text{(S5)}$$

exhibiting the novel term (n_D^2/λ) for surface plasmons in comparison to the common relationship n_D/λ for the polaritonic-like modes along the light cone. At SPR, its wavenumber position K_∞ is additionally strongly increased at the low magnitude ε_2«1 ("undamped SPR) by the factor $\left(\frac{1}{\sqrt{\varepsilon_2}}\right)$. This means high-wavenumber, short-wavelength plasmons can be efficiently excited in spectral ranges far from the strong interband absorption bands in the photoexcited material and, in semiconductors and dielectrics, at moderately high EHP densities, since both interband and and intraband transitions strongly contribute to the magnitude ε_2, Indeed, at high magnitudes $\varepsilon_2 \gg |\varepsilon_1|, \eta_1 > 1$ ("damped SPR), for $b \gg c$ one has the common relationship [12]

$$K_{SPP} = \frac{1}{\lambda}\sqrt{\frac{b+\sqrt{b^2+c^2}}{2a}} \approx \frac{\sqrt{\eta_1}}{\lambda} \approx \frac{n_D}{\lambda}, \quad \text{(S6)}$$

representing the low-wavenumber (n_D/λ), near-wavelength surface polariton-like waves. Similarly, under off-resonance, undamped SPP excitation conditions along the light cone – for $\varepsilon_1 \ll -\eta_1$, $\eta_2 \approx 0$ and $\varepsilon_2 \ll 1$

$$a = \varepsilon_1^{\,2}, \quad b = \eta_1\varepsilon_1^2, \quad c \approx \varepsilon_2\eta_1^2 \approx 0, \quad \text{(S7)}$$

and

$$K_{SPP,1} \approx \frac{1}{\lambda}\sqrt{\eta_1} = \frac{n_D}{\lambda}, \quad \text{(S8)}$$

where the derived solution exactly represents the low-wavenumber (n_D/λ), near-wavelength surface plasmon-polariton waves [10].

The prompt dielectric function of the photoexcited material was modeled as a function of electron-hole plasma (EHP) density ρ_{eh} and optical frequency Ω in the form [13]

$$\varepsilon^*_M(\Omega,\rho_{eh}) = \varepsilon_M(\Omega)\left(1-\frac{\rho_{eh}}{\rho_{sat}}\right) - \frac{\Omega_{PL}^2(\rho_{eh})}{\Omega^2+\nu(\rho_{eh})^2}\left(1-\frac{i\nu(\rho_{eh})}{\Omega}\right) \quad \text{(S9)},$$

where the plasma frequency $\Omega_{PL}(\rho_{eh})$ and scattering rate $\nu(\rho_{eh})$ were evaluated as follows [3,7,14]

$$\Omega_{PL}^2(\rho_{eh}) = \frac{\rho_{eh}e^2}{\varepsilon_0\varepsilon_{\mathrm{hf}}(\rho_{eh})m_{opt}^*}, \nu(\Omega,\rho_{eh}) = \left(\frac{\pi^2\sqrt{3}}{128E_F^{\,2}}\right)\frac{(\pi k_B T_e)^2+(\hbar\Omega)^2}{1+\exp\left(\frac{-\hbar\Omega}{k_B T_e}\right)}\Omega_{PL}(\rho_{eh}) \propto C\Omega_{PL}(\rho_{eh}), \quad \text{(S10)}$$

accounting for the effective optical e-h pair mass m_{opt}^*, the high-frequency dielectric constant $\varepsilon_{hf}(\rho_{eh})$, which due to EHP screening tends to 1 at near-critical EHP densities $\rho_{eh} \sim \rho_{crit}$ ($\rho_{crit} \approx 5\times10^{21}$ cm^{-3} in CLN at 1030 nm) defined from Equation (S9) as $\Omega_{PL}(\rho_{crit}) = \sqrt{\varepsilon(\Omega)}\,\Omega$, EHP saturation density for interband transitions ρ_{sat}, temperature T_e and Fermi level E_F, the numerical factor C ~10 in different dielectrics [3,6-9] (in crystalline CLN C ~10).

References

1. Raether, H. "Surface plasmons on smooth and rough surfaces and on gratings." Springer, Berlin (1988).
2. M. Shinoda, R. R. Gattass, and E. Mazur, Shinoda, Masataka, Rafael R. Gattass, and Eric Mazur, Femtosecond laser-induced formation of nanometer-width grooves on synthetic single-crystal diamond surfaces, Journal of Applied Physics, 105(5), (2009) 053102.
3. Danilov, P. A., Ionin, A. A., Kudryashov, S. I., Makarov, S. V., Rudenko, A. A., Saltuganov, P. N., Seleznev, L. V., Yurovskikh, V. I., Zayarny, D. A., Apostolova, T. "Silicon as a virtual plasmonic material: Acquisition of its transient optical constants and the ultrafast surface plasmon-polariton excitation." *Journal of Experimental and Theoretical Physics* 120(6) (2015): 946-959.
4. E. Rebollar, J.R. Vázquez de Aldana, J.A. Pérez-Hernández, T.A. Ezquerra, P. Moreno, and M. Castillejo, Ultraviolet and infrared femtosecond laser induced periodic surface structures on thin polymer films, Applied Physics Letters 100(4), (2012) 041106.
5. R. Buividas, L. Rosa, R. Šliupas, T. Kudrius, G. Šlekys, V. Datsyuk, and S. Juodkazis, Mechanism of fine ripple formation on surfaces of (semi) transparent materials via a half-wavelength cavity feedback, Nanotechnology 22(5) (2010) 055304.
6. S.I. Kudryashov, L.V. Nguyen, D.A. Kirilenko, P.N. Brunkov, A.A. Rudenko, N.I. Busleev, A.L. Shakhmin, A.V. Semencha, R.A. Khmelnitsky, N.N. Melnik, I.N. Saraeva, A.A. Nastulyavichus, A.A. Ionin, E.R. Tolordava, and Y.M. Romanova, Large-scale

laser fabrication of anti-fouling Si surface nanosheet arrays via nanoplasmonic ablative self-organization in liquid CS2 tracked by sulfur dopant, ACS Applied Nano Materials 1(6) (2018) 2461-2468.
7. S.I. Kudryashov, S.V. Makarov, A.A. Ionin, C.S.R Nathala, A. Ajami, T. Ganz, A. Assion, and W. Husinsky, Dynamic polarization flip in nanoripples on photoexcited Ti surface near its surface plasmon resonance, Optics letters 40(21) (2015) 4967-4970.
8. Kudryashov, S. I., Danilov, P. A., Rupasov, A. E., Smayev, M. P., Kirichenko, A. N., Smirnov, N. A., ... Zakoldaev, R. A. "Birefringent microstructures in bulk fluorite produced by ultrafast pulsewidth-dependent laser inscription." *Applied Surface Science* 568 (2021): 150877.
9. Kudryashov, S., Rupasov, A., Zakoldaev, R., Smaev, M., Kuchmizhak, A., Zolot'ko, A., ... Shur, V. "Nanohydrodynamic Local Compaction and Nanoplasmonic Form-Birefringence Inscription by Ultrashort Laser Pulses in Nanoporous Fused Silica." *Nanomaterials* 12(20) (2022): 3613-3622.
10. J. Gottmann, D. Wortmann, M. Horstmann-Jungemann, Fabrication of sub-wavelength surface ripples and in-volume nanostructures by fs-laser induced selective etching, Appl. Surf. Sci. 255 (10) (2009) 5641-5646.
11. Bell, R. J., Alexander, Jr., R. W., Parks, W. F., Kovener, G. "Surface excitations in absorbing media". *Optics Communications* 8(2) (1973): 147-150.
12. Kudryashov, S. I., Nastulyavichus, A. A., Saraeva, I. N., Rudenko, A. A., Zayarny, D. A., Ionin, A. A. "Deeply sub-wavelength laser nanopatterning of Si surface in dielectric fluids: Manipulation by surface plasmon resonance." *Applied Surface Science* 519 (2020): 146204.
13. Sokolowski-Tinten, K., von der Linde, D. "Generation of dense electron-hole plasmas in silicon." *Physical Review B* 61(4) (2000): 2643-2650.
14. A.A. Ionin, S.I. Kudryashov, S.V. Makarov, A.A. Rudenko, P.N. Saltuganov, L.V. Seleznev, and E.S. Sunchugasheva, Femtosecond laser fabrication of sub-diffraction nanoripples on wet Al surface in multi-filamentation regime: High optical harmonics effects? Applied Surface Science, 292 (2014) 678-681.
